# Chemoproteomics, A Broad Avenue to Target Deconvolution

**DOI:** 10.1002/advs.202305608

**Published:** 2023-12-14

**Authors:** Yihui Gao, Mingzhe Ma, Wenyang Li, Xiaoguang Lei

**Affiliations:** ^1^ Beijing National Laboratory for Molecular Sciences Key Laboratory of Bioorganic Chemistry and Molecular Engineering of Ministry of Education College of Chemistry and Molecular Engineering Peking University Beijing 100871 China; ^2^ Peking‐Tsinghua Center for Life Sciences Peking University Beijing 100871 China; ^3^ Academy for Advanced Interdisciplinary Studies Peking University Beijing 100871 China; ^4^ Institute for Cancer Research Shenzhen Bay Laboratory Shenzhen China

**Keywords:** chemical probe, chemoproteomics, forward chemical genetics, target identification

## Abstract

As a vital project of forward chemical genetic research, target deconvolution aims to identify the molecular targets of an active hit compound. Chemoproteomics, either with chemical probe‐facilitated target enrichment or probe‐free, provides a straightforward and effective approach to profile the target landscape and unravel the mechanisms of action. Canonical methods rely on chemical probes to enable target engagement, enrichment, and identification, whereas click chemistry and photoaffinity labeling techniques improve the efficiency, sensitivity, and spatial accuracy of target recognition. In comparison, recently developed probe‐free methods detect protein‐ligand interactions without the need to modify the ligand molecule. This review provides a comprehensive overview of different approaches and recent advancements for target identification and highlights the significance of chemoproteomics in investigating biological processes and advancing drug discovery processes.

## Graphical abstract

Striving to identify the molecular targets of a bioactive compound, target deconvolution acts as the link between phenotypic chemical screening and comprehensive exploration of the underlying mechanisms. Various chemoproteomic approaches, facilitated by chemical probe‐assisted target enrichment or probe‐free methodologies, have been developed and employed in target deconvolution. These approaches significantly contribute to elucidating biological mechanisms and advancing drug discovery processes.

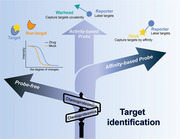

## 1 Introduction

**Figure 1:**
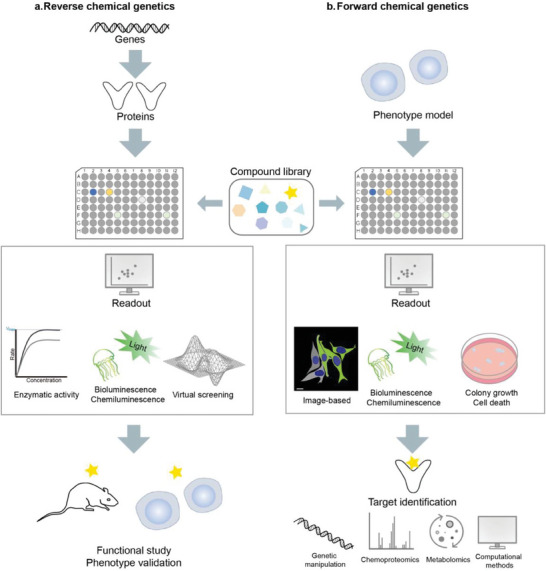
Reverse chemical genetics (a) versus forward chemical genetics (b). a) In reverse chemical genetics, genes or proteins of interest are screened with a compound library. Detectable readouts include, for example, the progression or rates of enzymatic reactions, bioluminescence and chemiluminescence. Virtual screening can also be performed, in which changes in energy may reflect the effects of the analyzed compounds. These methods provide hit compounds for further validation. After hits validation and structure‐activity relationship analysis, the lead compound is further utilized for functional studies and bioactivity measurements. b) In forward chemical genetics, phenotypical screenings begin in a model system to find candidate compounds. High‐content imaging, bioluminescence, chemiluminescence and the growth state of living systems may serve as readouts to assess the effects of tested compounds. The lead compounds undergo target deconvolution by genetic approaches, chemoproteomics, metabolomics or computational analysis, and are subsequently validated through biochemical means.

Chemical genetics is a multidisciplinary field that focuses on understanding the genomic and proteomic responses of biological systems using small molecule probes.^[^ ^1^ ^]^ It serves as a tight linker between library screening and genomic manipulations, providing powerful strategies to unravel biological pathways, identify drug targets, and develop new therapeutics. Chemical genetics can be categorized into two branches according to the starting point and the research process: the forward chemical genetics (**Figure** **1**a) and the reverse chemical genetics (Figure 1b).

The forward chemical genetics initiates with a chemical screening in a living biological system to observe the phenotypic responses.^[^ ^2^ ^]^ The screening assays often involve assessing protein activity, gene expression, or cellular behaviors. Once a positive compound with a desirable effect is identified, versatile profiling approaches are employed to explore its molecular targets and mechanism of action (MoA). In contrast with forward chemical genetics, which starts from phenotype, reverse chemical genetics focuses on specific genes or proteins of interest and aims to identify functional modulators for them to regulate and study cellular or organismal activities related with the target protein.

Both forward and reverse chemical genetics provide chemical compounds as valuable tools to dissect complex regulatory networks of genes, proteins, and biochemical pathways, as well as to explore the possibilities of targeting certain genes or pathways for the treatment of human diseases. In this context, we underline the contribution of forward chemical genetics in identifying novel druggable sites, gaining insights into biological processes, and advancing the development of new therapies.

The debate has persisted for several years in the pharmaceutical industry on whether target‐based (reverse chemical genetics) or phenotype‐based (forward chemical genetics) approaches are more effective for drug discovery.^[^ ^3^ ^]^ Conceivably, reverse chemical genetics that commences with a well‐defined gene or protein, facilitates the decipher of complex molecular interactions and provides means to dissect the contribution of individual genes or proteins to biological phenomena. Reverse chemical genetics approaches have successfully been applied to study many functional proteins, including GTPases, kinases, molecular motors, and receptors.^[^ ^4^ ^]^ Target‐based drug discovery avoids the difficulties in target deconvolution and enables the rational design and structure‐activity relationship (SAR) analysis of compounds. However, it suffers from poor productivity,^[^ ^5^ ^]^ incomplete insight of target, as well as poor translatability, as there could be disparities between molecular function and disease‐relevant phenotypes.^[^ ^3b^ ^]^ These limitations emphasize the significance of complementary phenotypic drug discovery, which excels in identifying novel drug leads with therapeutically relevant effects and molecular targets.^[^ ^3^, ^6^ ^]^ Forward chemical genetics, in the realm of drug exploration, takes into account the intricate interactions between multiple targets and pathways, making it particularly valuable for uncovering novel druggable targets and compounds with unique therapeutic effects.^[^ ^7^ ^]^ Taking a broader perspective, phenotypes interfered by drugs, metabolites, or natural products can be more easily observed through cell‐ or animal‐based assays, without limiting the readouts to the putative molecular targets‐related activities. However, the readout of cell‐ or animal‐based assays could be influenced by cellular uptake efficiency and bioavailability of compounds, leading to potential false negative results. Despite such drawbacks, the forward chemical genetic approaches offer advantages in terms of unbiased drug discovery and holistic investigation of complex biological systems. In this review, we underline the contribution of forward chemical genetics in identifying novel druggable sites, gaining insights into biological processes, and advancing the development of new therapies.

Target deconvolution plays a crucial role in forward chemical genetics, as it involves the identification and validation of specific genes, proteins, and pathways that are modulated by a given compound. In pharmacological study, target identification enables structure‐based approaches for lead optimization and provides explanations of drug side effects.^[^ ^8^ ^]^ By elucidating the target, researchers can potentially unravel novel MoA and corresponding biological pathways. In the field of personalized medicine, disclosing the specific targets underlying a disease allows scientists to customize precise treatments based on an individual genetic profile or unique expression patterns of target proteins in the patients.^[^ ^9^ ^]^

Commonly used approaches for target deconvolution include the genetic manipulations (transcriptome sequencing,^[^ ^10^ ^]^ RNAi,^[^ ^11^ ^]^ CRISPRi and CRISPRa^[^ ^12^ ^]^), metabolomic profiling,^[^ ^13^ ^]^ chemoproteomics,^[^ ^14^ ^]^ as well as knowledge‐based computational methods.^[^ ^15^ ^]^ Investigations based on genetic manipulation do not always phenocopy or recapitulate the effects of chemical leads, resulting in the disconnection between genetics data and subsequent development of inhibitors.^[^ ^14^, ^16^ ^]^ A few reasons may contribute to the discrepancies. Unlike chemical probes which may block one specific function of a protein, genetic depletion removes the entire protein and all its functions. The genetic gain or loss of function may activate compensatory mechanisms or redundant pathways in biological systems, masking the effects of targeted genes. Furthermore, some targets identified by genetic approaches are considered undruggable,^[^ ^17^ ^]^ such as transcription factors and the interfaces of protein–protein interactions. While metabolomics in target discovery faces challenges in clarifying the complex crosstalk between metabolites and the potential target, in silico methodologies are controversial in terms of reliability and accuracy. On the other hand, chemoproteomics focuses on proteins, the executors of cellular activities, providing a more straightforward and unbiased approach to mapping the target landscape. Assisted with high‐resolution mass spectrometry, chemoproteomics has significantly facilitated the profiling of drug MoA, characterization of binding specificity, and development of targeted therapeutics.

Identification and confirmation of bioactive targets have been compared to finding a needle in a haystack.^[^ ^18^ ^]^ The chemical probe can be likened to a magnet attracting the needle in classic chemoproteomics. In this review, we provide an overview of chemoproteomic approaches for target deconvolution. We present two types of experimental strategies classified as chemoproteomics utilizing chemical probes and newly developed probe‐free profiling. The probe‐based methodologies are described as those dependent on affinity‐based probes and those using activity‐based probes. We underline the role of classic probe‐based methods in systematic profiling, target identification, and characterization. Additionally, we summarize and emphasize the probe‐free methods as promising complement when effective probes are lacking.

## 2 Principles of Designing Chemical Probes

Chemical probes are a class of powerful tool molecules in chemical biology, that play an important role in various aspects, including exploring physiological and pathological processes, understanding drug MoA, expanding genome annotations, and identifying novel drug targets.

Genetic gain‐ and loss‐of‐function methods mediated by lentivirus, CRISPR/Cas9, and RNAi have provided significant insights into the physiological and pathological activities of proteins of interest in living organisms. Compared to genetic techniques that manipulate genes or gene expression, chemical probes have distinct advantages. Chemical probes modulate protein activity transiently and rapidly, rather than directly clearing or reducing the protein of interest. This temporal modulation allows for precise control and investigation of protein function. Although genetic manipulations often align with the modulations of small molecules phenotypically in many cases, there could be divergence due to the distinct intrinsic mechanisms. Certain genes possess redundant functions, making the phenotypic effects unclear when silencing a single gene. For different types of cells and tissues, the efficiency of genetic editing can be variable, for example, efficient and well‐tolerated genome editing in primary cells remains a major challenge.^[^ ^19^ ^]^ Additionally, the non‐specificity of lead compounds may interfere with the functions of several proteins, that can be challenging to include them all when engineering genes. Some proteins inhibited by compounds may also act as scaffolds for protein–protein interactions, which would be disrupted by genetic knockout or knockdown.^[^ ^20^ ^]^ Overall, these limitations of genetic manipulations and the gaps between genetic editing and chemical modulations make chemical probes complementary to genetic techniques well. Chemical probe toolbox acts as a bridge between biological phenotypes and specific molecular targets, providing insights into biological processes that may not be easily achievable through genetic approaches alone.

**Figure 2:**
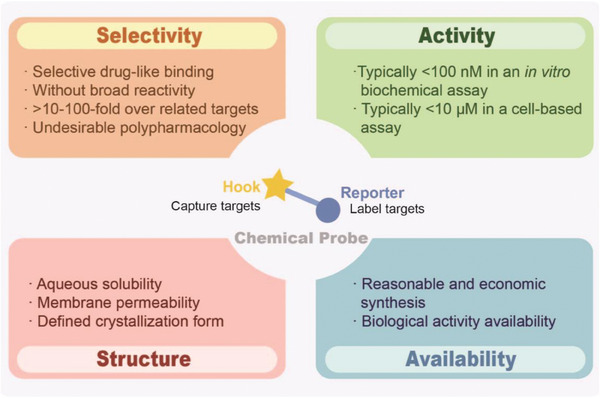
Key principles for designing and selecting effective chemical probes. Target selectivity is the prior principle in designing chemical probes. The high affinity, the reserved bioactivity, cell permeability and adequate solubility are crucial for effective target engagement. For experimental feasibility, structural and biological compatibility, as well as economic synthesis should be considered. These principles collectively ensure the efficacy and utility in complex biological system. These principles are summarized and modified from previous reviews.^[^ ^16^, ^21^ ^]^

Several reviews have summarized the characteristics of qualified chemical probes,^[^ ^16^, ^21^ ^]^ and the key features are outlined here (**Figure** **2**). High selectivity and potency are essential for a decent chemical probe. The concept named pan‐assay interference compounds (PAINS) defines a class of compounds with a wide range of biological activities. Deceptive phenotypes are often observed with PAINS, whose activities arise from non‐specific chemical reactions, chelating metal ions, or inducement of reactive oxygen species (ROS), but not from specific, druglike interactions between compounds and proteins.^[^ ^22^ ^]^ For example, quinones have a high propensity for being redox‐active and reactive toward nucleophiles present in the side chains of proteins, such as cysteine and lysine.^[^ ^23^ ^]^ Some compounds containing quinone structures, such as mitomycin C,^[^ ^24^ ^]^ doxorubicin,^[^ ^25^ ^]^ have been shown to exert complex biological effects by inducing ROS production. Protein kinases are key regulators of cellular function, and constitute one of the largest and the most functionally diverse protein families. The development of selective kinase inhibitors is challenging, because the ATP‐binding sites of kinase are relatively conserved.^[^ ^26^ ^]^ LDN192960 was developed initially as a Haspin inhibitor and was found to exhibit off‐target effects on DYRK and PIM isoforms.^[^ ^27^ ^]^ After kinase profiling was carried out, LDN192960 was found to inhibit DYRK isoforms (DYRK2, DYRK3, and DYRK1A) potently.^[^ ^28^ ^]^ Wei and coworkers have developed C17, a derivative of LDN192960, through multiple rounds of structure‐based optimization guided by several co‐crystallized structures.^[^ ^29^ ^]^ C17 displayed outstanding selectivity for the human kinome containing 467 other human kinases, which enables the identification of several novel DYRK2 targets.^[^ ^30^ ^]^ The poor selectivity of chemical probes may lead to ambiguous or misleading results because of the interactions with unintended targets. On the contrary, highly selective chemical probes help confirm the biological role of a specific target, avoiding potential interference from unrelated molecules. In addition to high selectivity, biological potency is also the basic requirement of chemical probes. Typically, the activities of probes are expected to be below 100 nm in an in vitro biochemical assay and below 10 µm in a mechanistic cell‐based assay.^[^ ^20^, ^21^ ^]^

Chemical properties of qualified probes impact on the exposure at sites of action. Two key factors in this regard are aqueous solubility and membrane permeability, which are especially important for research at the cellular or organismal levels.^[^ ^31^ ^]^ Since the biological systems mainly consist of water environments, good aqueous solubility allows a probe to be easily administered and interact with biological targets. Membrane permeability means probe has to pass through the cellular membrane barriers to reach its target. Membrane permeability is related to the physiochemical properties of chemical probes such as the charge, lipophilicity, and size. In addition, chemical availability and public‐associated data of a probe could greatly facilitate follow‐up studies and peer evaluations. Chemical availability refers to the ease with which the probes are synthesized or modified. Furthermore, the accessibility of public data of probes enables research peers to replicate and validate the results, thereby facilitating transparency, reproducibility and peer evaluation. In brief, high selectivity, potent bioactivity, and appropriate physicochemical properties, as well as their availability in terms of synthesis and inclusion in databases, contribute to the utility of probes and the foundations for further evaluations.

## 3 Probe‐Based Chemoproteomics

### 3.1 Affinity‐Based Protein Profiling (AfBP)

Direct chemoproteomics methods utilize bifunctional chemical probes that consist of a bioactive moiety for capturing the target proteins and a derived handle for subsequent visualization or enrichment. The “bait” used to capture target proteins from cell extracts or live cell systems can be categorized into two types based on its interaction with the target: affinity‐based probes (which rely on potent noncovalent interactions) and activity‐based probes (which involve the covalent interactions). Here, we introduce some representative studies (**Table** **1**) taking advantage of AfBP for target identification.

**Table 1:** Examples using affinity‐based probes for target deconvolution.

Molecule	Bioactivity	Probe	Target identified	Refs.
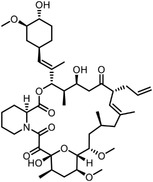	Immunosuppressive	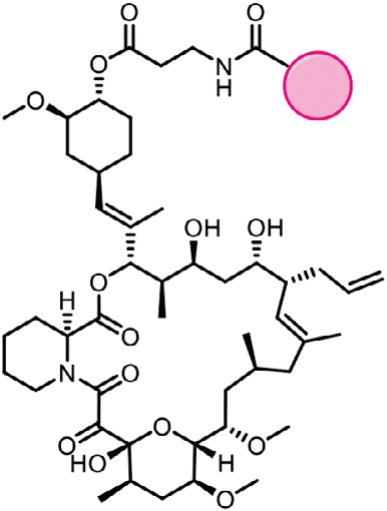	FK506‐binding proteins (FKBPs)	[32]
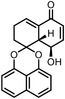	Anti‐tumor	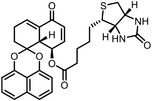	Proteasome activator PA28 gamma (PA28γ)	[34]
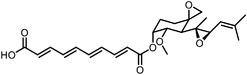	Anti‐angiogenic	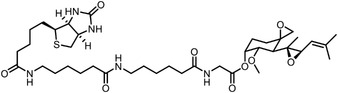	Methionine aminopeptidase 2 (MetAP‐2)	[35a]
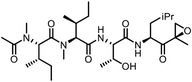	Anti‐tumor, anti‐inflammatory	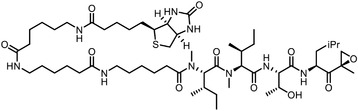	Proteasome	[35b]
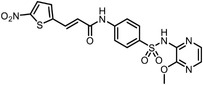	Blocking necrosis	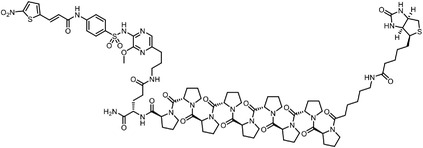	Mixed lineage kinase domain like pseudokinase (MLKL)	[36]
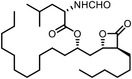	Anti‐obesity, antitumor	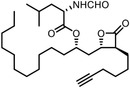	Fatty acid synthase (FAS) (expected), eight new targets	[40]
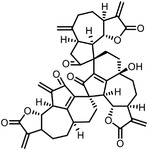	Anti‐cancer	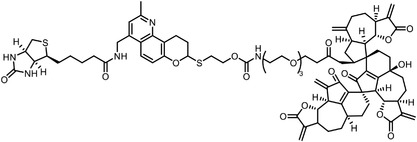	Peroxisome proliferator‐activated receptor gamma (PPARγ)	[42]
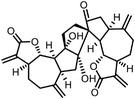	Inhibiting NF‐κB signaling pathways	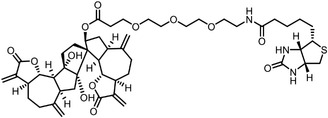	I kappa B kinase (IKKα/β)	[43]
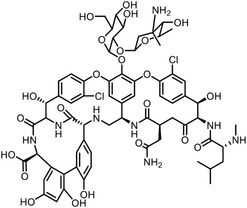	Inhibiting the bacteria cell wall biosynthesis	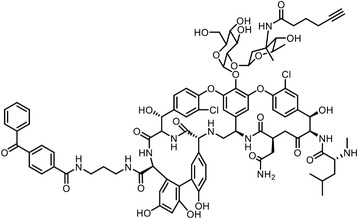	Staphylococcal autolysin Atl and an ABC transporter protein in *E. faecalis*	[50]
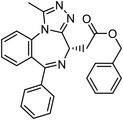	BRD4 inhibitor as an epigenetic regulator	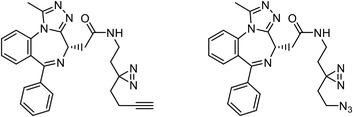	Apurinic/apyrimidinic endonuclease 1 (APEX1) (off‐target)	[51]
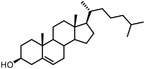	Component of cell membrane and precursor for several other signaling molecules	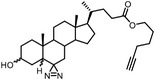	Over 250 candidate proteins, including receptors, channels and enzymes	[55]
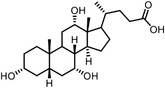	Endogenous metabolite and anti‐bacteria	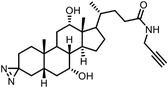	Histidine kinases (HKs) of the two‐component systems (TCS) in bacteria	[56]
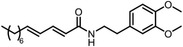	Inhibitor of AEA (Anadamide or N‐arachidonoylethanolamine) re‐uptake	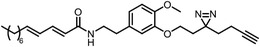	Saccharopine dehydrogenase‐like oxidoreductase (SCCPDH), vesicle amine transport 1 (VAT1), and ferrochelatase (FECH)	[58a]
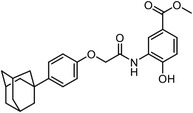	Inhibiting HIF‐1α accumulation	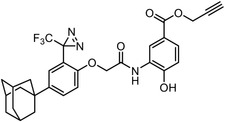	Malate dehydrogenase 2 (MDH2)	[58b]
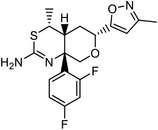	BACE1 inhibitor	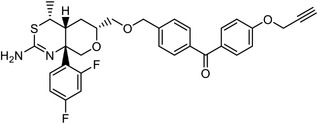	Cathepsin D (CatD) (off‐target)	[58c]
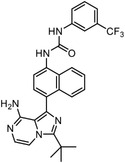	Inositol‐requiring enzyme 1α (IRE1α)	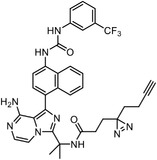	Several nucleotide‐binding proteins	[58d]
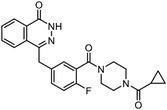	Poly (ADP‐ribose) polymerases (PARPs) inhibitor	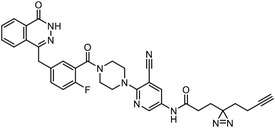	Several off‐target candidates	[58e]
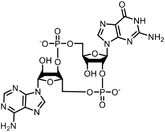	Innate immune response	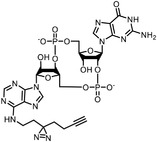	Eukaryotic translation elongation factor 1 alpha 1 (EF1A1)	[59b]
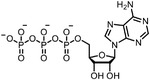	Basic cell metabolite and signaling molecule	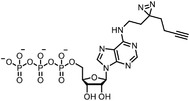	FAS receptor, CD44, and various SLC transporters	[59c]
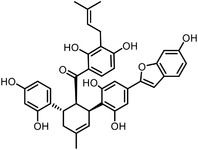	A plant natural product, anti‐microbial	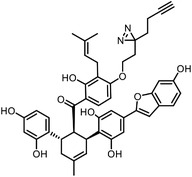	*Morus alba* Diels–Alderase (MaDA)	[60]
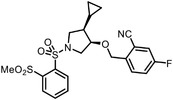	Activating the secretion of ApoE	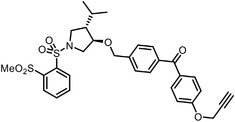	Liver X receptor beta (LXRβ)	[61a]
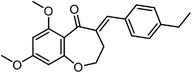	Anti‐inflammatory	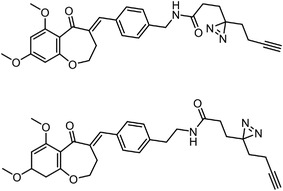	Pyruvate kinase muscle isozyme M2 (PKM2)	[61b]

**Figure 3:**
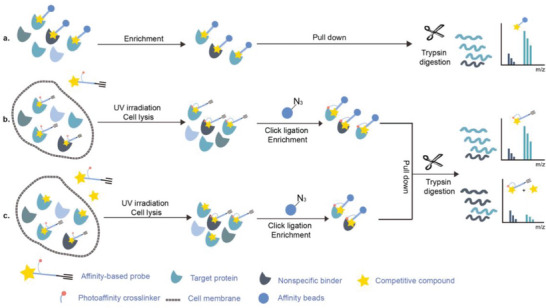
General schemes of affinity‐based protein profiling. a) The compounds can be immobilized on a solid support, to directly pull the interacting proteins down. The modified version (b,c) uses probes with photoaffinity tags for covalently crosslinking, and clickable handles for ligation with affinity matrix. c) To exclude the false positives, original compounds are added as competitors to occupy the genuine binding sites of probes. Enriched proteins can be trypsinized for further MS detection.

Early research of affinity‐based target discovery has identified that FKBP is a target of the immunosuppressive macrolide FK506. With FK506 immobilized matrix (**Figure** **3**a), target proteins were pulled down in lysates from bovine thymus and from human spleen.^[^ ^32^ ^]^ The biotin‐streptavidin interaction, one of the most potent non‐covalent interactions, is widely used for affinity enrichment.^[^ ^33^ ^]^ Rhytidenone F is a natural product that possesses an unsaturated ketone moiety, which is a good Michael acceptor that can form covalent bonds with nucleophilic residues of proteins. Utilizing the biotinylated Rhytidenone F as a positive probe and a reduced negative control probe, Yue et al. have identified PA28γ as the direct target of Rhytidenone F, which further leads to antitumor effects.^[^ ^34^ ^]^ Natural products with reactive unsaturated ketone moieties can tightly capture the interacting proteins, that can be easily pulled down on beads. The Crews group incubated sample lysates with biotinylated natural products, followed by streptavidin agarose purification, successfully fishing out the covalently interacting proteins with covalently reactive compounds.^[^ ^35^ ^]^ With that strategy, they have identified a metalloprotease, methionine aminopeptidase (MetAP‐2) as a binding protein of the anti‐angiogenic agent fumagillin.^[^ ^35a^ ^]^ Moreover, they have proven that epoxomicin acted as an antitumor agent by specifically targeting proteasome.^[^ ^35b^ ^]^ To boost the capacity of purification, Sun et al.^[^ ^36^ ^]^ introduced a polyproline‐rod spacer between the biotin tag and a necrosulfonamide lead, inspired by the work of Sato and coworkers.^[^ ^37^ ^]^ Within this biotinylated necrosulfonamide probe, they discovered that necrosulfonamide directly interacts with the mixed lineage kinase domain‐like protein (MLKL), blocking necrosis downstream of RIP3 activation.

Advances in click chemistry have provided powerful tools in synthesizing derivatives or mimics of drugs, natural products and pharmacophores.^[^ ^38^ ^]^ Direct biotinylation of probes can introduce a large chemical group that may impair biological activity and complicate the synthesis process. Therefore, once the drug compound or lead compound of interest has been identified, it is preferred to introduce a bioorthogonal group to the compound to enable an unbiased proteomic study and facilitate the assessment of its cellular selectivity.^[^ ^16^ ^]^ Clickable handles are often designed to be very small, in order to minimize their possible impacts on the compounds’ bioactivity and selectivity. Among the various bioorthogonal reactions, the copper‐catalyzed union of azide and alkyne to form triazole is widely used, due to its high bio‐compatibility and specificity.^[^ ^39^ ^]^ After target engagement, biotin or Rhodamine can be easily appended to the probe via the 1,3‐dipolar cycloaddition of azides and alkynes, supporting further enrichment or visualization respectively.

Click chemistry is particularly helpful when conducting in situ (in living systems rather than in lysates) chemoproteomics, due to its stable performance across complex biological environments.^[^ ^38^ ^]^ The incubation of lysates with chemical probes suffers from some disadvantages, for example: the spatial‐resolved information could be lost, weak binders are often excluded when using non‐covalently interacting probes, and the biological relevance is reduced compared to in situ labeling. To address these limitations, probes can be incorporated with intrinsically covalently reactive groups or photoreactive groups. Intrinsically, covalently reactive groups encompass Michael receptors with conjugated unsaturated double‐bond structures, or substrate analogs that are recognized and catalyzed for covalent conjugation by endogenous enzymes.

Orlistat is an approved anti‐obesity drug and a promising antitumor drug. To explore the off‐targets and side effects of Orlistat, Yang et al. have designed several probes containing the β‐lactone for target capturing and a terminal alkyne group for click derivation.^[^ ^40^ ^]^ In addition to the expected fatty acid synthase (FAS), they have identified a total of eight new targets of Orlistat, in which HSP90AB1 may partially account for the antitumor effect of this drug. Our group have established a new biorthogonal ligation termed TQ ligation, enabled by click hetero‐Diels‐Alder (HDA) cycloaddition of *o*‐quinolinone quinone methides and vinyl thioethers.^[^ ^41^ ^]^ Using this highly selective ligation technique, we reported a novel anticancer mechanism of (‐)‐ainsliatrimer A, which contains four α, β‐unsaturated ketones as Michael acceptors that can react with other biomolecules.^[^ ^42^ ^]^ Further pull‐down assay and biochemical validations revealed that PPARγ is a functional cellular target of (‐)‐ainsliatrimer A. Using a similar designing strategy, IKKα/β were identified as the functional targets of ainsliadrimer A, resulting in the inhibition of NF‐κB signaling pathways.^[^ ^43^ ^]^

Metabolic labeling allows probes to be metabolically incorporated into target biomolecules, with the help of endogenous or modified enzymes, such as, the glycan transferases^[^ ^44^ ^]^ and the acyl transferases.^[^ ^45^ ^]^ In two recent studies, Wen and Zhou et al. exploited a sensitive and reversible probe for core fucosylation,^[^ ^46^ ^]^ and then improved it for labeling core fucosylation and *O*‐GlcNAcylation using two mutant endoglycosidases.^[^ ^47^ ^]^ In the latter case, they also introduced a temperature‐sensitive resin to capture and enrich the labeled glycoproteins by manipulating the temperature. The wild‐type endoglycosidases can recognize and release the captured glycopeptides enzymatically, allowing further MS analysis in a traceless manner.^[^ ^47^ ^]^ Metabolic labeling offers relatively firm capture of target proteins of the tested metabolites, but is limited by the substrate compatibility of endogenous enzymes and is not suitable for exploring non‐covalent and weak ligand‐target interactions.

Photoaffinity labeling (PAL) is a straightforward and unbiased strategy that generates highly reactive transient species to crosslink the probe to proximal biomolecules upon irradiation with specific wavelength^[^ ^48^ ^]^ (Figure 3b). Arylazides, benzophenones, and diazirines, especially the trifluoromethyl derivative, are commonly used photoaffinity groups for labeling biomolecules. However, arylazides have fallen out of favor due to their potential damage to biomolecules when excited in situ. Benzophenones can be activated with a harmless long wavelength of irradiation, and show inertness to the solvent.^[^ ^49^ ^]^ For instance, Eirich et al. have revealed that the potent antibiotic vancomycin has another two non‐classical targets, Staphylococcal autolysin Atl and an ABC transporter protein in *E. faecalis*, through using bifunctional vancomycin‐based probes, containing an azide clickable handle and a benzophenone group for photo‐crosslinking.^[^ ^50^ ^]^ Diazirines, small photoaffinity groups, are commonly employed as they generate suitable mimics of the original compounds. Upon irradiated, diazirines generate carbenes that rapidly crosslink with biomolecules. Pan et al. have developed alkyl diazirine‐containing photo‐crosslinkers with various biorthogonal tags.^[^ ^51^ ^]^ Utilizing these crosslinkers, they modified an inhibitor of BRD4, GW841819X, and then discovered its novel off‐target, APEX1.

**Figure 4:**
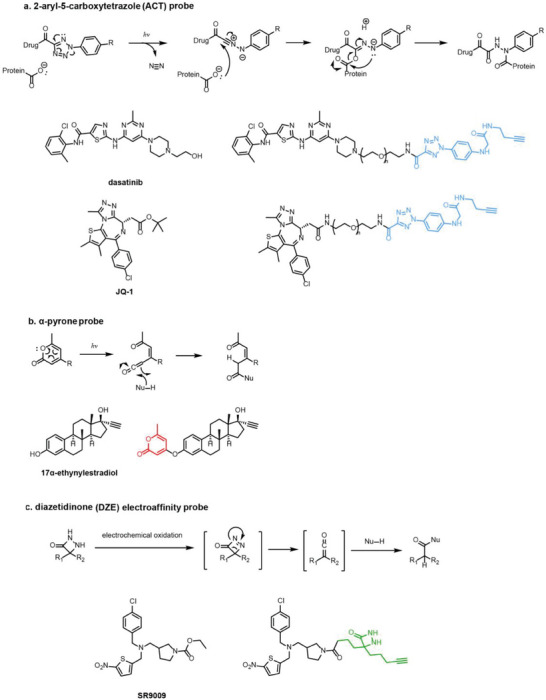
Recently developed affinity‐based labeling chemistry. a) The 2‐aryl‐5‐carboxytetrazole (ACT) photoaffinity label was developed by A. Herner et al.,^[^ ^52^ ^]^ and was applied in dasatinib‐based and JQ‐1 based probes. The blue‐colored structures indicate the photoaffinity reactive groups and clickable handles. b) The α‐pyrones photoaffinity label was developed by O. A. Battengerg et al.,^[^ ^53^ ^]^ and such strategy had been applied in several different probes such as a 17α‐ethynylestradiol based probe. The red‐colored structure indicates the photoaffinity reactive group. c) The diazetidinone electrochemical label was developed by Y. Kawamata et al.,^[^ ^54^ ^]^ which can be electrochemically oxidized and produce reactive intermediate in biocompatible conditions. Chemoproteomics profiling of SR9009 was performed in such electroaffinity labelling (ECAL) way. The green‐colored structure indicates the electroaffinity reactive group and a clickable handle.

Besides the canonical PAL strategies involving arylazides, benzophenones, and diazirines, novel photo‐initiated chemical labeling techniques have been developed. 2‐Aryl‐5‐carboxytetrazole (ACT) is a new photoaffinity label that undergoes nucleophilic addition to the carboxy‐nitrile imine followed by the acyl shift to generate specific photoadducts.^[^ ^52^ ^]^ Compared to canonical photoaffinity labels, ACT has shown higher yields in the ligand‐directed photo‐cross‐linking reactions with the recombinant target proteins and enabled the in situ target detection in a similar manner with diazirine (**Figure** **4**a). α‐Pyrones and Pyrimidones could also serve as photoaffinity probes, such modifications were further applied in studying the interactome of 17α‐ethynylestradiol in *E. coli* ^[^ ^53^ ^]^ (Figure 4b).

Photoaffinity labeling strategies use high‐energy ultraviolet irradiation, which generates reactive moieties to complicate downstream analysis. Recently, Baran and coworkers have developed an electroaffinity labeling platform that leverages the use of a small, redox‐active diazetidinone functional group.^[^ ^54^ ^]^ Diazetidinone can be electrochemically oxidized to covalently crosslink with its direct interacting partners in living cells, which opens up new avenues for capturing targets (Figure 4c).

Benefiting from the progresses in quantitative mass spectrometry, many negative controls are introduced to stratify true target proteins, for instance, competing the true‐positive interactions with excess free compound (Figure 3c), labeling without irradiation or using probes without clickable handles. The Cravatt group have combined clickable, photoreactive sterol probes with quantitative mass spectrometry to globally discover potential cholesterol‐binding proteins.^[^ ^55^ ^]^ In this research, excess cholesterol occupied genuine binding sites competitively to reduce non‐specific binding, while stable‐isotope labeling by amino acids in cell culture (SILAC) mass spectrometry method was used for further quantitative analysis. This approach also leads to the profiling of the interactors of bile acids.^[^ ^56^ ^]^ Different from C‐6‐derived cholesterol probes, photoaffinity modifications of bile acid probes were introduced at the C‐3 position of the steroid core, suggesting that a case‐by‐case analysis is needed when designing chemical probes. Quantitative chemoproteomics can be combined with the fragment‐based ligand and drug discovery (FBLD), enabling the proteome‐wide FBLD in human cells.^[^ ^57^ ^]^ Photoaffinity and bioorthogonal groups were embedded into the fragments common to many drug structures to compose a library of fully‐functionalized‐fragment probes, which can be further optimized into higher‐affinity ligands.

Affinity‐based proteomics profiling is a powerful tool for illustrating the MoA or side‐effects of drugs,^[^ ^58^ ^]^ discovering the sensing pathways of endogenous molecules,^[^ ^59^ ^]^ identifying the substrates of enzymes,^[^ ^60^ ^]^ fishing the target proteins in phenotypic screenings.^[^ ^61^ ^]^ When assessing the functional state of proteins in cells or tissues under certain conditions, activity‐based protein profiling (ABPP) may provide a suitable alternative as discussed in the next section.

### 3.2 Activity‐Based Protein Profiling (ABPP)

Protein expression, modification, localization, and interactions are highly dynamic. Under the regulation of various signals, the levels and the subcellular localizations of different proteins vary greatly. Proteins have abundant post‐translational modifications (PTMs), such as phosphorylation and ubiquitination, which significantly regulate protein homeostasis and activity. Some existing methods such as multidimensional liquid chromatography coupled with tandem mass spectrometry (LC/LC‐MS/MS),^[^ ^62^ ^]^ yeast two‐hybrid (Y2H) experiment^[^ ^63^ ^]^ and protein microarray,^[^ ^64^ ^]^ are successful in exploring protein functions from the aspects of PTMs and interactions, but are not suitable for the direct evaluation of protein activity.

ABPP is a straightforward method that uses active site‐directed covalent probes, the activity‐based probes (ABPs), to characterize protein function directly in native biological systems on a global scale.^[^ ^65^ ^]^ The bifunctional ABPs possess one reactive group that covalently labels the active site and one reporter tag for visualization or enrichment. The reactive group exploits reactivity or conserved mechanistic/ structural features of active sites to catch functional enzymes. To target enzymes presenting nucleophilic residues in their active pockets, electrophilic reactive groups are designed. For instance, serine hydrolase contains a conserved serine nucleophile in the catalytic core, which could be susceptible to covalent modifications by fluorophosphonates^[^ ^66^ ^]^ and aryl phosphonate electrophiles.^[^ ^67^ ^]^

**Figure 5:**
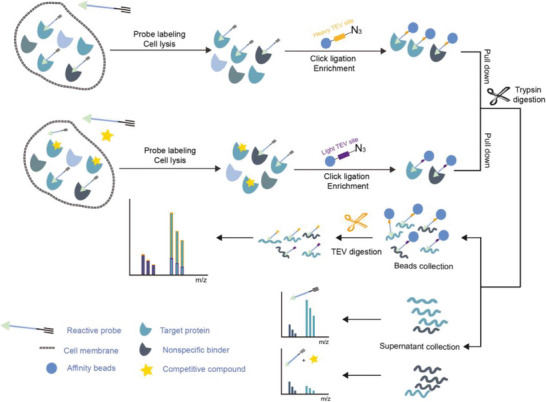
General schemes of affinity‐based protein profiling. a) Compounds can be immobilized on a solid support, to directly pull the interacting proteins down. The modified version (b,c) uses probes with photoaffinity tags for covalently cross‐linking, and clickable handles for ligation with affinity matrix. c) To exclude the false positives, original compounds are added as competitors to occupy the genuine binding sites of probes. Enriched proteins can be trypsinized for further MS detection.

The platforms utilizing ABPP in target identification have experienced multiple rounds of optimizations and developments. The earliest version is gel‐based ABPP, which was developed by Benjamin F. Cravatt^[^ ^66^ ^]^ and Matthew Bogyo^[^ ^68^ ^]^ in the late 1990s. In this approach, protein extracts are incubated with functional probes, followed by gel separation and visualization. The intensity of the bands corresponds to the level of enzyme activity in samples. Gel electrophoresis platforms are convenient and robust for rapid comparative analysis, but they are limited by the low resolution. When combined with LC‐MS, ABPP approach can further distinguish proteins with similar biophysical properties in 1D or 2D polyacrylamide gel electrophoresis (PAGE). In 2005, the Cravatt group combined the ABPP and multidimensional protein identification technologies (ABPP‐MudPIT),^[^ ^69^ ^]^ in which enzyme activity signatures are categorized with in‐gel fluorescence analysis, followed by affinity enrichment and on‐bead digestion before the in‐depth LC‐MS/MS analysis. When digesting the peptides on beads, the probe‐labeled peptides cannot be released from the beads, therefore, information on the specific labeling sites is not available. An improved method, termed active‐site peptide profiling (ASPP), was exploited, in which digestion with trypsin is prior to affinity enrichment.^[^ ^70^ ^]^ ASPP provides some information on the identity of interacting peptides at the cost of compromising the coverage of enriched proteins. TOP (tandem‐orthogonal proteolysis)‐ABPP^[^ ^71^ ^]^ was developed to characterize the labeled proteins and the modified sites in parallel. TOP‐ABPP introduces a tobacco etch virus (TEV) protease cleavage site inserted between the reactive group and the reporter group of chemical probes. After labeling with probes, target proteins can be captured by the affinity tags, followed by a tandem digestion with trypsin and TEV protease. The reactive peptides can then be identified with greatly reduced false‐positive signals given by unspecific absorption of affinity matrix. Conceivably, quantitative mass spectrometry methodologies can be readily combined with TOP‐ABPP, leading to the development of the isoTOP‐ABPP approach (**Figure** **5**). Weerapana and coworkers have first described isoTOP‐ABPP in 2011, to profile quantitatively the intrinsic reactivity of cysteine residues en masse directly in native biological systems.^[^ ^72^ ^]^ In addition to synthesizing the isotopically labeled iodoacetamide‐alkyne probes, isobaric tags for relative and absolute quantitation (iTRAQ), tandem mass tag (TMT) label and SILAC are commercially available labeling techniques for quantitative MS analysis. Limited by the high cost of these reagents, Wang group came up with an affordable and conveniently accessible approach called rdTOP‐ABPP,^[^ ^73^ ^]^ where the trypsinized peptides were isotopically derivatized by triplex reductive demethylation, respectively. In the discovery of inhibitors, ABPs can bind to the reactive pockets of proteins to hinder the access of inhibitors to the substrates. Inspired by this cognition, competitive ABPP method was reported for discovery of reversible enzyme inhibitors, avoiding extensive protein expression, protein purification, and establishment of specific substrate assays.^[^ ^74^ ^]^

Data‐dependent acquisition (DDA) and data‐independent acquisition (DIA) are two major MS acquisition schemes. In the DDA mode, the precursor ions from a survey scan are selected and fragmented sequentially based on their abundance,^[^ ^75^ ^]^ thus, more of the low‐abundance peptides might be missing. While in the DIA mode, all precursors contained in the predetermined isolation windows are scanned and fragmented,^[^ ^76^ ^]^ providing higher quantitative accuracy and reproducibility. Recently, the Wang group have established the DIA‐ABPP platform for comprehensive profiling of functional cysteineome,^[^ ^77^ ^]^ opening up a new opportunity for in‐depth and multi‐dimensional profiling of functional proteomes in complex biological systems.

Since information about protein activity can be obtained directly using ABPP, it has become a major approach for enzyme inhibitor discovery and function assignment, including developing selective inhibitors of a specific target, and exploring ligand‐protein interactions broadly and agnostically.^[^ ^78^ ^]^ In the context of forward chemical genetics strategies, ABPP can be applied to conveniently analyze the target engagement and the off‐targets of a given compound, in living cells or in vivo.

Carbamates are a group of versatile serine hydrolases (SH) inhibitors, based on which Cravatt et al. have generated a library of SH‐directed inhibitors, which could be used to systematically develop selective inhibitors against uncharacterized serine hydrolases.^[^ ^79^ ^]^ Through screening on this carbamate library combined with competitive ABPP, they developed a potent and selective inhibitor of ABHD6. Because the ABPP chemistry often targets numbers of enzyme families, the strategy in which ABP labeling is competed out by enzyme inhibitors, is widely utilized.^[^ ^80^ ^]^ An elegant case used such competitive ABPP to explain the clinical neurotoxicity of BIA 10–2474, an inhibitor of the fatty acid amide hydrolase (FAAH).^[^ ^81^ ^]^ By mapping the interacting protein landscape of BIA 10–2474 in human cells and tissues, researchers attempted to understand the potential of promiscuous lipase inhibitors to cause metabolic dysregulation in nervous system. The competing agents are not limited to existing reversible compounds, but can also be covalent ligands derived from de novo design or modular synthesis. Through screening a synthesized covalent ligand library using ABPP strategies for functional allosteric methionine, key methionine sites were disclosed and a potential inhibitor of CDK4 was proposed.^[^ ^82^ ^]^

For a conserved enzyme family, it is not difficult to develop a broad‐spectrum inhibitor. The kinases perform their function of phosphorylation using ATP as a co‐substrate, leading to a classical avenue to develop ATP analogs as kinase inhibitors. Nevertheless, the comparable inhibitory mechanisms shared by most kinase inhibitors necessitate a high level of selectivity in drug discovery, which poses a formidable challenge for reverse chemical genetics screening.^[^ ^83^ ^]^ Kinobeads is one tool derived from competitive ABPP,^[^ ^84^ ^]^ for immobilizing broad‐spectrum kinase inhibitors on matrix, to quantify the potency and selectivity of potential inhibitors. An alternative method, termed KiNativ, constructs ABPs comprised of ATP linked to biotin through an acyl‐phosphate bond.^[^ ^85^ ^]^ With this platform, kinase inhibition could be quantified as competitive reductions by affinity enrichment. Recently, with considerations that the reactive lysines of target proteins show pronounced accessibility and activity changes in response to ABPs, Hao et al. introduced a target‐responsive accessibility profiling (TRAP) approach.^[^ ^5^, ^9^ ^]^ With TRAP system, they have mapped numbers of responsive target candidates of several major glycolytic metabolites, which inspires the further exploitation of activity‐based target deconvolution.

Chemocentric high‐throughput screening (HTS) is a useful drug discovery tool, from which the lead compound will undergo complicated target identification and validation through omics or biochemical approaches. Backus and coworkers have simplified the HTS with covalent fragment‐based ligand discovery (FBLD), and then combined a cystine‐reactive FBLD with isoTOP‐ABPP.^[^ ^86^ ^]^ This improvement provides a powerful platform for the discovery of novel covalent ligands and facilitates the exploration of the functions and interactome of emerging protein targets.

## 4 Probe‐Free Chemoproteomics

Probe‐based chemoproteomics requires high selectivity and potency of functionalized small molecules, which might be challenging and time‐consuming to achieve. Natural products often have novel chemical structures and great drug potential, however, their complicated skeletons pose big challenges to synthesis and modification. Probe‐independent approaches that detect target candidates of small molecules are emerging in recent years. These probe‐free methods are designed to measure changes on the biophysical properties of proteins, such as thermal stability,^[^ ^87^ ^]^ rates of oxidation,^[^ ^88^ ^]^ and stability of proteins from proteolysis^[^ ^89^ ^]^ and chemical denaturation,^[^ ^90^ ^]^ based on the assumption that these properties of proteins are changed upon binding to the ligand.

**Figure 6:**
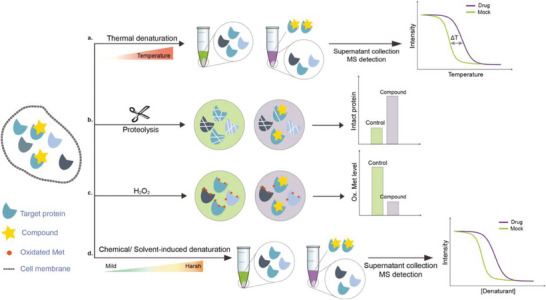
Schematic diagram of probe‐free chemoproteomics approaches in target identification. The biophysical properties of proteins, including a) thermal stability, b) susceptibility to proteolysis, c) the rate of oxidation and d) the resistance to chemical denaturation, change upon ligand binding. The purple lines or light purple bars indicate the drug‐treated samples. The green lines and light green bars indicate the mock control.

Changes in protein conformation occur upon ligand binding,^[^ ^91^ ^]^ which in turn may alter its thermal stability. The thermally induced unfolding of a protein often demonstrates a sigmoidal melting curve, giving a characteristic melting temperature (Tm),^[^ ^92^ ^]^ which may change when a ligand is bound to the protein. When heated, many proteins in cells or cell lysate become unstable and rapidly precipitate. The remaining soluble proteins can be quantified to provide corresponding melting curves. Molina et al. reported the first cellular thermal shift assay (CETSA) in 2013, a broadly applicable assay to validate drug targets, as well as to monitor the process of drug transport and activation, off‐target effects, and drug resistance.^[^ ^87^ ^]^ Assisted with quantitative mass spectrometry, the thermal stability curves and the ligand‐induced shifts are obtained to reveal the possible drug mechanisms (**Figure** **6**a). Savitski et al. have performed thermal proteome profiling (TPP) both on intact cells and cell extracts, through which they could distinguish if the effects are induced by direct ligand binding or by modifications of downstream effectors.^[^ ^93^ ^]^ The early TPP protocols used buffer without detergent to lyse samples, which provide limited information about transmembrane proteins. To overcome this shortcoming, mild detergents are used during cell lysing in recently developed protocols.^[^ ^94^ ^]^ Taking advantage of the powerful quantitative mass spectrometry, the Savitski group have established a comprehensive proteomic off‐target profiling platform, combining a two‐dimensional TPP (drug concentration and temperature) with affinity enrichment‐based chemoproteomics.^[^ ^95^ ^]^ The 2D‐TPP strategy enabled a more sensitive identification for the marketed HDAC inhibitor Panobinostat, providing possible explanations for the adverse effects observed in clinic. Bergamini and coworkers further expanded the applications of TPP to the blood and tissue levels, and drew the organ‐specific, proteome‐wide thermal stability maps.^[^ ^96^ ^]^ Moreover, because mild thermal treatment might induce allosteric changes without completely denaturing proteins, Leuenberger et al. have profiled protein thermostability on a proteome‐wide scale using limited proteolysis (LiP), providing insights into the molecular and evolutionary bases of protein and proteome thermostability at a domain‐level resolution.^[^ ^89b^ ^]^

In the decade since the birth of TPP, quite a lot of molecular targets of drugs and metabolites have been identified with TPP.^[^ ^94^, ^97^ ^]^ The phenotypic HTS assay on natural products generates positive compounds that are often difficult to be synthesized or modified. As a new target deconvolution strategy complementary to classical probe‐based chemoproteomics, TPP (MS‐CETSA) has enabled the identification for many complex natural products or metabolites, such as NPD10084,^[^ ^98^ ^]^ quinine^[^ ^99^ ^]^ and Vioprolide A.^[^ ^100^ ^]^ However, this approach is limited by the extent of structural alternations of target proteins.^[^ ^89b^ ^]^ The binding strength of the ligand to the receptor is not directly proportional to the magnitude of the thermal stability of target proteins. In some cases, the induced changes in thermal stability might be very small, even for tightly bound ligands,^[^ ^94a^ ^]^ leading to false negatives. TPP in intact cells might also detect the post‐translational modifications, protein–protein interactions, and protein degradations, which increases the false positive rate of analysis.

The ligand‐induced stabilizations include not only the stabilities to heat denaturation, but also the proteolysis (Figure 6b), oxidative denaturation (Figure 6c), and chemical denaturant (Figure 6d). When bound by ligand, protein might become less susceptible to proteolysis,^[^ ^101^ ^]^ which was exploited by the Huang group for target deconvolution,^[^ ^102^ ^]^ giving rise to a new method termed DARTS (drug affinity responsive target stability). The Huang group has applied DARTS to reveal the potential molecular target and mechanism of action of resveratrol, a natural product in red grapes. With the DARTS approach, the modulation of clathrin activity by lathyrane diterpene was identified.^[^ ^103^ ^]^ Qu et al. focused on the Wnt (wingless)/β‐catenin signaling pathway and combined DARTS with 2D‐DIGE (two‐dimensional difference in gel electrophoresis), identifying the E3 ubiquitin ligase SHPRH as the direct target of axitinib in blocking Wnt/β‐catenin signaling.^[^ ^104^ ^]^ A similar method was utilized to disclose the peptidyl arginine deiminase 2 (PAD2) as the functional target of nitazoxanide for Wnt inhibition.^[^ ^105^ ^]^ Recently, the Qu group identified a natural product liquidambaric acid as an inhibitor of Wnt signaling by phenotype‐based screening, and uncovered its target protein TRAF2 by DARTS.^[^ ^106^ ^]^

Fitzgerald et al. introduced the SPROX (stability of proteins from rate of oxidation) approach that utilizes hydrogen peroxide in the presence of increasing concentrations of a chemical denaturant to oxidize proteins. They confirmed with two model protein systems, that the presence of ligands influences the rates of oxidation of target proteins, corresponding precisely with the reported Kd values. SIP^[^ ^90a^ ^]^ (solvent‐induced protein precipitation) and CPP (chemical denaturation and protein precipitation)^[^ ^90b^ ^]^ respectively used guanidine hydrochloride or organic solvent to induce protein precipitation, which yielded better proteomic coverage than that produced by SPROX. Besides of those derivatization‐free chemoproteomics based on the responses of receptors to proteolysis or denaturing stress, metabolites are utilized as triggers of the alternations in the proteolytic resistance of proteins. Geiger and coworkers discovered that elevating l‐arginine levels induce global metabolic changes in T cells and mouse models, by integrated analysis on proteome and metabolome.^[^ ^107^ ^]^ Changes due to metabolite‐protein interactions can be further amplified by LiP,^[^ ^89b^ ^]^ and then measured by high‐resolution MS.^[^ ^108^ ^]^ Picotti et al. applied such workflow to map the metabolite‐protein interactome, shed light on the prevalence and mechanisms of enzyme promiscuity, and enabled extraction of quantitative parameters of metabolite binding on a proteome‐wide scale.^[^ ^108^ ^]^

The probe‐free methods for target identification avoid limitations imposed by narrow chemical modification windows, save the cost on probe retrofitting, and lower the barrier of target deconvolution, especially for natural products. However, the above‐mentioned approaches detect peptides after denaturation, resulting in the low abundance and coverage of proteome. This limitation poses a big challenge to the detection of low‐abundance genuine targets. In contrast, probe‐based methods utilize affinity enrichment to increase the relative concentration of the target proteins in samples tested. More sensitive equipment, enhanced depth of proteome coverage, and further optimized protocols may compensate this deficiency to a certain extent.

## 5 Outlooks and Perspectives

Chemoproteomics plays a pivotal role in target discovery, providing valuable insight into ligand‐protein interactions, which helps unravel the mechanisms of drug action. Among the versatile chemoproteomic tools (**Table** **2**), affinity‐based protein profiling and activity‐based protein profiling rely on the precise design and synthesis of chemical probes, which then allows the selective engagement, enrichment, identification, and quantification of potential target proteins. In the exploitation of chemical probes, click chemistry and photoaffinity labeling greatly facilitate the in situ labeling and the subsequent affinity enrichment, improving the spatial accuracy of target recognition, as well as elucidating the specific binding sites. There are many derived approaches from classical AfBP or ABPP, shedding light on protein abundance, subcellular localizations, interactions, and post‐translational modifications. There are also many modified versions of chemoproteomic methods that enhance the throughput or reduce experimental cost. Nevertheless, these probe‐dependent approaches are limited by the complicated SAR analysis, probe design, and synthesis process, leading to poor efficiency and productivity. The non‐classical chemoproteomics independent on probe‐labeling has developed rapidly in recent years. These probe‐free methods detect ligand‐protein interactions based on the altered biophysical properties upon ligand binding, including changes in thermal stability, resistance to oxidation, proteolysis, or chemical stress. Of note, approaches without probe‐labeling save time and cost, but the current protocols still suffer from low proteomic coverage and relatively high false‐positive or false‐negative rates.

**Table 2:** Comparison of chemoproteomic methods for target identification.

Methods	Chemical probes	Features	Limitations
AfBP	Affinity matrix‐immobilized pull‐down	Compounds immobilized on matrix or beads; incubation with cell lysates	Not generally applicable; difficult to pick derivatization sites
	Affinity‐based pull‐down	Biotinylation of chemical probes	Complicated synthesis; large biotin group may affect bioactivity; tricky to design linkers
	Click chemistry‐enabled affinity‐based pull‐down	The affinity group is linked via click reaction; minimized alteration of bioactive compounds	Challenging and time‐consuming synthesis; loss of low‐affinity target information due to wash procedure;
	Photoaffinity probe‐based protein profiling	Suitable for in situ labeling; covalently capture targets	Nonspecific crosslinking; synthesis can be challenging and time‐consuming
ABPP	Gel‐based ABPP	Labeled proteins are separated in gel; convenient and robust	Low resolution
	ABPP‐MudPIT	In‐gel fluorescence analysis combined with LC‐MS/MS	Loss of the information on labeling sites
	ASPP	Digestion with trypsin is prior to affinity enrichment	Low protein coverage
	TOP‐ABPP	Combination of ABPP‐MudPIT and ASPP; labeled peptides are released by TEV digestion	Not quantitative
	isoTOP‐ABPP	Quantitative TOP‐ABPP	High cost in quantitative tags
	rdTOP‐ABPP	Peptides are isotopically derivatized by triplex reductive dimethylation; more affordable and convenient	Limited to triplex quantification
	DIA‐ABPP	MS data acquisition in DIA mode; higher quantitative accuracy and reproducibility; enhanced depth of profiling	Difficult data processing
CETSA/TPP	Probe‐free	Protein thermal stability changes upon ligand binding, reflected in protein abundance and melting temperature	Low protein coverage; expensive labeling reagents
DARTS		Protein stability against proteolysis increases upon ligand binding, reflected in protein abundance	False negative and false positive
SPROX		Protein stability against oxidation changes upon ligand binding, reflected in oxidized protein abundance	Not suitable for Met‐poor proteins; false negative; low protein coverage
CPP/SIP		Protein stability against chemical denaturation changes upon ligand binding, reflected in protein abundance	Low protein coverage; expensive labeling reagents

Despite the challenges mentioned above, there is an increasing number of success examples that demonstrate how chemoproteomics assist in target identification. Besides further enhancing the sensitivity, resolution, accuracy, and speed of MS analysis and quantification, the improvements in data acquisition and analysis algorithms will provide more accurate interpretation of proteomics data, while a novel chemical toolbox will expand the scope of proteins’ dynamic function map. Different chemoproteomic tools can complement with each other to uncover more comprehensive information on ligand‐protein interactions. Furthermore, chemoproteomics can be integrated with multi‐omics technologies (transcriptomics, metabolomics), to foster a wider recognition on biological effects of small molecules. Systematically combining these optimized approaches not only facilitates the deconvolution of target proteins, the exploration of various biological processes, but also has profound implications for better understanding disease progression and developing new therapeutic interventions.

## Conflict of Interest

The authors declare no conflict of interest.
